# Pharmacological and non-pharmacological treatments for opioid-induced constipation: A protocol for systematic review and network meta-analysis

**DOI:** 10.1097/MD.0000000000014161

**Published:** 2019-01-25

**Authors:** Jing Zhang, Lanfang Mao, Longde Wang, Cuncun Lu, Xiaojuan Du, Qiankun Liang, Bo Yang, Hongli Wu

**Affiliations:** a Gansu University of Traditional Chinese Medicine; b Affiliated Hospital of Gansu University of Traditional Chinese Medicine; c Evidence-Based Medicine Center, School of Basic Medical Sciences, Lanzhou University, Lanzhou, China.

**Keywords:** acupuncture, network meta-analysis, opioid-induced constipation, pharmacological treatment, systematic review

## Abstract

**Background:**

Pain is very common and its management with a huge burden for patients and the healthcare system. And the network meta-analysis was designed to provide reference for the clinical practice.

**Methods:**

PubMed, EMBASE, Cochrane library, CNKI, VIP, Wan Fang, and CBM will be systematically searched their inception to November 2018. Randomized controlled trials that compared the effect of differently pharmacological or non-pharmacological treatments for opioid-induced constipation will be included. The primary outcome is the efficacy of therapeutic regimens. Risk of bias assessment of the included studies will be performed using the Cochrane risk of bias tool. A network meta-analysis will be performed using STATA 13.0 software with WinBUGS 1.4.3 software. Grading of Recommendations Assessment, Development, and Evaluation will be used to assess the overall quality of evidence.

**Results:**

This study is ongoing and will be submitted to a peer-reviewed journal for publication.

**Conclusion:**

This study will provide a comprehensive evidence on the effectiveness and safety of pharmacological and non-pharmacological treatments for opioid-induced constipation.

**PROSPERO registration number:**

CRD42018116533.

## 1 Introduction

Pain is very common and its management with a huge burden for patients and the healthcare system,^[1,2]^ and opioids are always prescribed to patients suffered from pain in clinical practice.^[2,3]^ Recently, opioid-induced adverse effects, especially, constipation, have led to great concern for their using because of inadequately counsel.^[4]^ Therefore, studies related opioid-induced constipation are increasingly, correspondingly, many pharmacological and non-pharmacological interventions including naloxone, alvimopan, methylnaltrexone, subcutaneous, and herb medicine, acupuncture and massage, single or combination used to treat opioid-induced constipation. Although some studies^[5–7]^ have discussed the question, their relative efficacy and safety are always no unclear. For example, Luthra et al^[6]^ found naloxone and naldemedine are the most effective treatments, naloxone was the safest agent, but they did not include traditional Chinese medicine and studies written with Chinese.

However, comprehensive and detailed search for all the available eligible studies is the cornerstone of the systematic review, and the issue has been discussed a long time, the only 1 effective method may be cooperating with non-native speaking groups to overcome language obstacle.^[8,9]^

Therefore, we designed a network meta-analysis to compare all pharmacological and non-pharmacological treatments for opioid-induced constipation and fill the gap. And we hope the results of the study will provide a reference for clinicians and improve patient's quality of life.

## 2 Methods

### 2.1 Eligibility criteria

#### 2.1.1 Type of study

Randomized controlled trials that investigated the effect of pharmacological or non-pharmacological treatments for the treatment of opioid-induced constipation will be included in the network meta-analysis.

#### 2.1.2 Type of patients

Adults (aged 18 years or older) with opioid-induced constipation, they were diagnosed based on the history of constipation associated with opioid analgesic use. There are no limitations in age, gender, race, or nationality.

#### 2.1.3 Type of interventions

One or combination of pharmacological (eg, methylnaltrexone, oxycodone, and Chinese herb medicine, etc, including placebo) or non-pharmacological (eg, acupuncture, massage, and enemata, etc) treatments to compare others therapies. There are no limitations in form, dose, and duration, and so on.

#### 2.1.4 Type of outcomes

The primary outcome is efficacy, including the disappearance or significant improvement in opioid-induced constipation. The secondary outcome is an overall adverse event, including diarrhea, nausea and abdominal pain, and so on. Randomized controlled trials reporting on at least one above situation will be included. And efficacy or adverse effect must be assessed with binary data.

### 2.2 Data source

We will search 7 electronic databases from their inceptions to November 2018, including PubMed, EMBASE, Cochrane library, CNKI, VIP, Wan Fang, and CBM. The search strategy of PubMed was as follows:

#1 “Constipation”[Mesh] OR “Gastrointestinal Transit”[Mesh] OR constipation [Title/Abstract] OR “gastrointestinal transit”[Title/Abstract] OR “slow transit”[Title/Abstract]
#2 “Opiate Alkaloids”[Mesh] OR “Analgesics”[Mesh] OR “Analgesics, Opioid”[Mesh] OR opiate [Title/Abstract] OR opiates [Title/Abstract] OR analgesics [Title/Abstract] OR opioid [Title/Abstract] OR opioids [Title/Abstract]
#3 “Randomized Controlled Trial” [Publication Type] OR “Controlled Clinical Trial” [Publication Type] OR random∗[Title/Abstract]
#4 #1 AND #2 AND #3

### 2.3 Study selection

All records will be loaded from electronic databases and inputted into EndNote X9 software to remove duplicate and check their eligibility. The screening process including 2 stages, first, 2 authors will independently check the title and abstract of all citations according to our eligibility criteria, second, potentially relevant studies also will be loaded for further assessment to ensure all available studies are included. And any discrepancy will ask the third reviewer to make the final decision. The Microsoft Excel 2016 will be used to design a data-abstract form, 5 eligibility studies will be used to test its property, then will revise it and collect relevant information. The abstracted information, including first author, publication year, sample size, intervention details, and relevant outcomes.

### 2.4 Risk of bias of individual studies

Two authors will independently evaluate the risk of bias for all included studies using the risk of bias's tool,^[10]^ including 6 domains: random sequence generation, allocation concealment, blind, incomplete outcome data, selective reporting, and other bias. And any discrepancy will through discussion or asking the third author to reach the agreement.

### 2.5 Statistical analysis

#### 2.5.1 Pairwise meta-analyses

The pairwise meta-analyses will be performed using STATA 13.0 software. The relative risk (RR) with 95% confidence interval (95% CI) will be used to measure outcomes. And random effects model will be used to pool effect estimate. The potential heterogeneity across all eligibility studies will be tested using *I*^2^. If the *P* value < .1 and *I*^2^ > 50%, we will explore sources of heterogeneity by subgroup analysis. Publication bias will be tested using Egger test^[11]^ through STATA 13.0 software when at least included 10 studies for 1 outcome.^[12]^

#### 2.5.2 Network meta-analyses

The STATA 13.0 software and WinBUGS 1.4.3 software will be used to perform a Bayesian network meta-analysis. The outcomes will be reported as RR with 95% CI. The node splitting method will be used to test inconsistency between direct and indirect comparisons.^[13]^ Surface under the cumulative ranking area ^[14]^ will be used to rank the different therapeutic regimen. Network geometry will use nodes torepresent different treatments and edges to represent the head-to-head treatments. And the size of node represents sample sizes of treatments, thickness of edge represents numbers of included studies.^[15]^

### 2.6 Quality of evidence

The quality of evidence of outcomes will be assessed using the Grading of Recommendations Assessment, Development, and Evaluation,^[16,17]^ and it including 5 degrade factors for randomized controlled trials, including risk of bias, inaccuracy, inconsistency, indirectness, publication bias, and results of assessment will be graded 4 levels: high level, moderate, low, and very low.

## 3 Discussion

To the best of our knowledge, this is the first network meta-analysis to assess pharmacological and non-pharmacological treatments for opioid-induced constipation. Although there are similar studies have discussed the question, they did not include Traditional Chinese medicine, also did not include papers written with Chinese. Therefore, the results of this study will fill the gap for the field and will provide the reference for clinical practice. Eventually, we will report the network meta-analysis according to the PRISMA extension statement for network meta-analyses.^[18]^

## Author contributions

**Conceptualization:** Lanfang Mao, Longde Wang.

**Data curation:** Jing Zhang, Xiaojuan Du, Qiankun Liang, Hongli Wu.

**Funding acquisition:** Longde Wang.

**Methodology:** Jing Zhang, Lanfang Mao, Cuncun Lu.

**Resources:** Longde Wang.

**Software:** Cuncun Lu, Xiaojuan Du, Qiankun Liang.

**Supervision:** Bo Yang, Hongli Wu.

**Validation:** Cuncun Lu, Bo Yang, Hongli Wu.

**Visualization:** Qiankun Liang.

**Writing – original draft:** Jing Zhang.

**Writing – review and editing:** Jing Zhang, Lanfang Mao.
